# Obituary

**Published:** 1890-04

**Authors:** 


### Obituary.

Dr. W. T. King, of Shelbyville, Ind., passed into the mysterious beyond on December 7th, after an illness of a few weeks with typhoid fever. The deceased located in the above place in 1880, succeeding Dr. J. S. Rice. He was well and favorably known as an able and conscientious dentist, of which fact there was no better evidence than his prosperous and increasing business. He shunned professional notoriety, but the few who knew him well realize that the profession has lost one of its brightest minds and a professional example worthy of emulation. Peace to his ashes.

